# Physical education teaching mode assisted by artificial intelligence assistant under the guidance of high-order complex network

**DOI:** 10.1038/s41598-024-53964-7

**Published:** 2024-02-19

**Authors:** Xizhong Song

**Affiliations:** Physical Education Center, Xijing University, Xi’an, 710123 China

**Keywords:** High-order complex networks, Artificial intelligence assistant, Physical education, Tennis teaching, Fuzzy cognitive map, Computer science, Information technology

## Abstract

This study explores the integration of artificial intelligence (AI) teaching assistants in sports tennis instruction to enhance the intelligent teaching system. Firstly, the applicability of AI technology to tennis teaching in schools is investigated. The intelligent teaching system comprises an expert system, an image acquisition system, and an intelligent language system. Secondly, employing compressed sensing theory, a framework for learning the large-scale fuzzy cognitive map (FCM) from time series data, termed compressed sensing-FCM (CS-FCM), is devised to address challenges associated with automatic learning methods in the designed AI teaching assistant system. Finally, a high-order FCM-based time series prediction framework is proposed. According to experimental simulations, CS-FCM demonstrates robust convergence and stability, achieving a stable point with a reconstruction error below 0.001 after 15 iterations for FCM with various data lengths and a density of 20%. The proposed intelligent system based on high-order complex networks significantly improves upon the limitations of the current FCM model. The advantages of its teaching assistant system can be effectively leveraged for tennis instruction in sports.

## Introduction

An increasing number of educational disciplines are incorporating artificial intelligence (AI) technology to enhance teaching effectiveness, driven by the continuous advancement of this technology. In the realm of physical education, AI sports technology is widely utilized, rendering physical education more intelligent and individualized to better cater to the diverse needs and traits of students^1–3^. An emerging sports training approach rooted in AI is referred to as the intelligent sports training system. These systems can formulate customized training schedules for students based on their physical characteristics, athletic abilities, and training requirements. Additionally, they can monitor students’ sporting progress in real-time, offering timely advice and insights. The integration of AI technology into sports equipment gives rise to intelligent sports equipment^4^. By tracking and analyzing students’ sporting activities in real-time, these systems provide individualized and scientifically informed sports advice and training plans.

AI in the educational landscape has the potential to automate grading processes and offer feedback to students through AI teaching assistant systems, thereby enabling educators to allocate their time to other responsibilities^5,6^. However, as AI applications expand, expert systems encounter challenges in handling complexities. Presently, artificial neural networks (ANNs) are confined to shallow models, limiting their efficacy in addressing intricate issues and utilizing vast datasets for learning and training efficiently. Managing and operating complex AI systems pose difficulties for traditional big data or massive network systems, as well as conventional analysis and optimization techniques. The extensive search space and complex spatial patterns of optimization problems further complicate the modeling of vast and intricate systems. In this context, the fuzzy cognitive map (FCM), as an intelligent computing tool, stands out for its advantages in abstractness, flexibility, and fuzzy reasoning, making it an ideal choice for system modeling and reasoning when compared to standard techniques like expert systems and neural networks^7^. In recent years, researchers have explored the application of AI techniques, such as neural networks and Bayesian networks, in education. For instance, neural networks are utilized to develop intelligent teaching systems that adapt to students’ needs autonomously. Juan et al.^8^ (2021) emphasized that neural network models could self-learn and adjust based on student performance and feedback, thereby offering a more personalized teaching experience. Additionally, Bayesian networks have found application in education, particularly in knowledge reasoning and decision-making. Nyberg et al.^9^ (2022) asserted that Bayesian networks were employed to analyze common fallacies in informal logic, study and evaluate various arguments, and expose common errors in evidence reasoning. Despite these advancements, the application of these techniques to physical education has received limited attention, with existing studies primarily focusing on theoretical exploration and model design. The challenges associated with large-scale complex system modeling and real-time stream data modeling remain unresolved using current FCM learning methodologies. Addressing these critical issues is paramount to enhancing the effectiveness of FCM in modeling complex systems.

In current educational technology, intelligent teaching systems predominantly rely on preset teaching plans and models, lacking the flexibility to adapt to individual differences and specific needs of students. This limitation arises from the systems’ emphasis on predetermined teaching strategies, overlooking the dynamic progress and distinct needs of students. Moreover, these systems often lack adaptability in responding to the intricate dynamics of the teaching process, hindering timely adjustments based on student performance and feedback. In addressing these challenges and recognizing the individual differences in students and the unique context of physical education, this study proposes an intelligent-assisted physical education teaching model based on high-order complex networks to enhance existing technology. The landscape of intelligent technology is evolving, particularly with the increasing integration of AI technology in sports. This evolution encompasses various aspects of computer vision, such as the introduction of eagle-eye technology in tennis. Complex networks are crucial tools and techniques for comprehending intricate systems, especially when seeking explanations for complex real-world scenarios. In the realm of advancing AI technology in tennis, this study endeavors to analyze the incorporation of AI technology into sports tennis instruction. Additionally, an intelligent modeling model is implemented to tackle real-world challenges. FCM serves as a modeling tool for complex systems in developing AI helper systems, and a more precise and reliable FCM model is proposed. The compressed sensing theory establishes a framework for learning large-scale FCM from time series data, and a framework for time series prediction based on high-order FCM is introduced.

This study makes a significant contribution by advancing the sophistication of physical education teaching models through the integration of AI assistance guided by high-order complex networks. Firstly, the research centers on the application of AI technology in school tennis instruction, constructing an intelligent teaching system comprising an expert system, an image acquisition system, and an intelligent language system. The design of this system is expected to elevate the intelligence level of tennis instruction. Secondly, the study employs compressive sensing theory to formulate a learning framework for a large-scale FCM, known as Compressive Sensing-FCM (CS-FCM). This framework aims to address challenges associated with automatic learning methods in the designed AI-assisted teaching system. This innovative approach provides a novel avenue for learning large-scale FCMs from time series data, offering theoretical support for the intelligence of teaching assistant systems. Lastly, the research proposes a time series prediction framework based on a high-order FCM. Through experimental simulations, CS-FCM demonstrates robust convergence and stability, providing crucial support for the development of intelligent systems and significantly improving the limitations of current FCM models. In summary, this study introduces AI-assisted teaching by incorporating guidance from high-order complex networks, presenting novel perspectives and methods for enhancing the intelligence of physical education teaching models. The positive results obtained in experimental simulations validate the effectiveness of the proposed model, laying the foundation for its broader application in future physical education teaching. This research contributes valuable experiences and references for the future integration of artificial intelligence in the field of education.

## Materials and methods

### AI technology in sports tennis teaching mode

**Figure 1:**
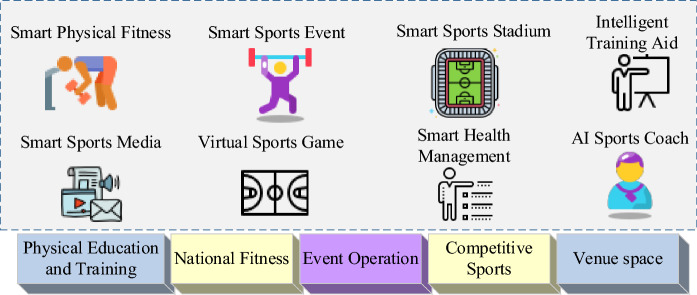
Intelligent application of sports events based on AI.

The market for AI in sports is projected to reach approximately 1.4 billion USD by 2020, with expectations for significant growth to 19.2 billion USD by 2030 at a compound annual growth rate of 30.3%^10^. The role of AI technology in sports continues to evolve, providing innovative and valuable contributions to sporting events. Two fundamental aspects of sports competitions are adherence to rules and engaging in competitive activities. The integration of AI technology with competitive events enhances training and facilitates advancements in the competition landscape. A critical challenge in competitive sports training lies in analyzing whether athletes’ training activities, including speed, angle, and strength, align with the project regulations promptly^11,12^. Figure 1 illustrates the intelligent application of AI-based sports events.

**Figure 2:**
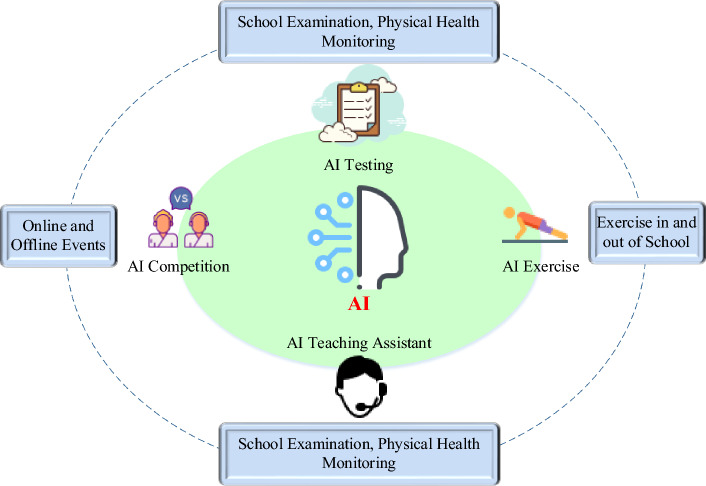
Four typical services of AI in physical education.

An increasing number of tennis teams and coaches are embracing the use of AI technology to enhance player training and management. Prominent teams, such as the French tennis team, leverage AI systems for analyzing game data and devising comprehensive training plans. Across various clubs, coaches, and teams, there is a growing trend of integrating AI technology to optimize training methods and analyze data for performance enhancement. However, as this technology continues to evolve, the specific details and applications are undergoing continuous refinement and updates, with no current unified standard. The integration of AI into competitive sports training involves utilizing cameras to capture athletes’ data, analyzing and assessing them through video-based human motion recognition algorithms, and drawing informed conclusions^13–15^. This approach allows athletes to gain intuitive insights into their training shortcomings and deficiencies, enabling them to receive higher-quality training plans through coach guidance and analysis. The four primary applications of AI in physical education are illustrated in Fig. 2. The intelligent AI sports teaching assistant system incorporates machine vision, AI cloud computing, and other technical means to generate personalized exercise prescriptions, provide targeted exercise suggestions, and establish a digital exercise space, creating an integrated closed loop of “learning, practicing, competing, and testing.”

In the realm of competitive sports, the construction of prediction models for athletes’ conduct often relies on extensive game films. Stanford University researchers have notably contributed to this domain by developing a statistical model of athletes’ behavior. Utilizing the marked hitting cycle database, encompassing a sequence of actions from preparation through hitting to recovery, the researchers generated players’ hitting and return decisions. This study introduces a mechanism for constructing actual video segment datasets, instrumental in creating controllable video systems that produce high-quality videos based on diverse real data^16^. The procedure employs the technique of neural image migration to mitigate variations in an athlete’s appearance (clothing, hairstyles, etc.) on different competition days or at various competition periods. Additionally, the algorithm addresses the challenge of missing pixel data when a player is only partially visible, ensuring the new video’s stability and coherence, such as the player’s stance in response to assessing the ball. The integration of data-efficient online optimization introduces an agent-assisted method that fosters a novel interaction between AI and athletes. Within the interactive cycle, athletes can select customized models with AI, establishing a feedback loop where training data and results can be iteratively incorporated into the model. This iterative process enhances the synergy between AI technology and athletes, contributing to a dynamic and adaptive training environment.

### Tennis instruction enhanced by an AI assistant system

In the contemporary era marked by the proliferation of AI technologies, encompassing 5G and big data, the education sector is compelled to seamlessly integrate education with AI technology. The deployment of AI as a teaching assistant has significantly amplified the efficacy of teaching methodologies, propelling the frontier of equitable education. Table 1 illustrates the specific applications of AI teaching assistant systems within the educational domain. AI assistant technology engages in analyzing and grading actions, concurrently providing technical support to students throughout their learning trajectories. Moreover, AI addresses the challenges faced by educators in thoroughly observing, providing nuanced feedback, and facilitating a profound understanding of the content. At the core of AI’s capabilities lies pattern recognition, a technique wherein computers emulate human senses to perceive and interpret data from the external environment. Propelled by the rapid evolution of digital information theory and computer technology, endeavors persist to evaluate natural information and convert it into digital signals. This evaluation enables the analysis and processing of digital signals through computational intelligence. A pivotal contribution of AI in education is the construction of virtual learning environments and data resources for educators. This project empowers teachers to meticulously plan lessons, organize classrooms, and prepare instructional materials in advance. By leveraging intelligent teaching tools and software, teachers can amass data on their students’ learning endeavors, facilitating sophisticated analyses at the teaching level to garner profound insights into the academic progress of their pupils.

**Table 1:** Specific application of AI teaching assistant system in the education industry.

Name of AI teaching assistant	Applicable field	Specific function
Assessments (America)	English comprehension, reading, and speaking	Formation of an audio-scoring system to assess students’ emotional engagement during reading
Interactive (America)	Physics, biology, computer, reading and writing	Language teaching, student understanding identification, and emotional feedback
Auto Tutor (America)	Physics, science, mathematics, reading and writing	Diagnosis of the learning process and provision of individualized guidance to students
Polaris (China)	All subjects	Assessment of students’ mastery of knowledge points, resource sharing, and testing of learning situations
101 education PPT	All subjects	Cloud storage resource database, teaching data recording, and learning situation analysis
Juku correction network	Composition writing	Correction of composition errors and sentence-by-sentence feedback

**Figure 3:**
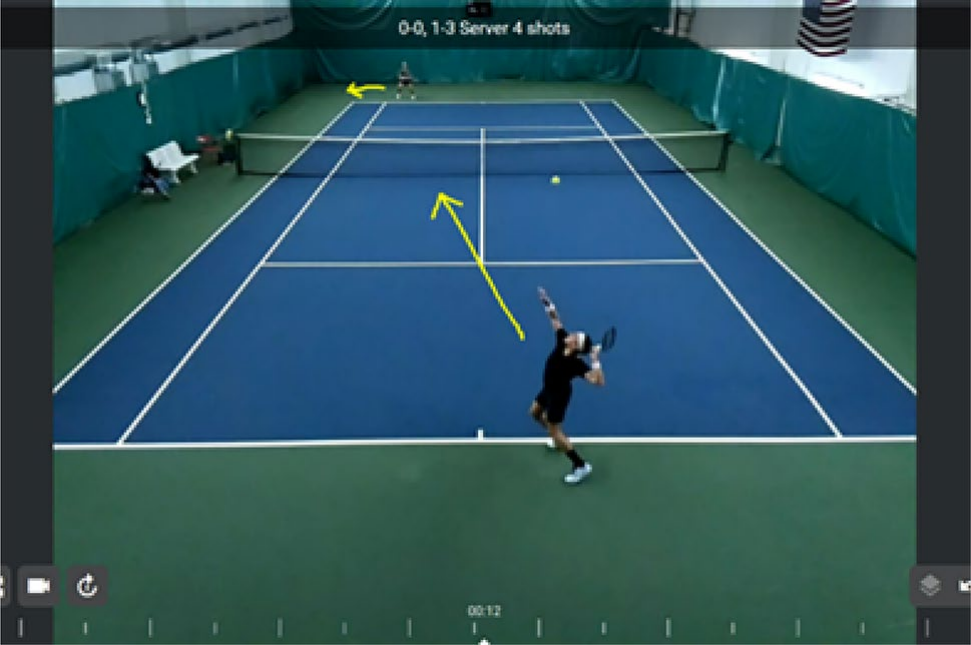
Real-time monitoring of serve training (Source: AccuTennis official website).

The AI assistant application’s visual recording feature enhances the training process’s clarity. This capability allows students and instructors to jointly review training videos, facilitating in-person discussions about areas that may need improvement in technical actions. Moreover, students can more effectively identify their shortcomings. Actively correcting errors becomes possible for students as they watch videos and follow the guidance provided by AI teaching assistants. An AI teaching assistant system utilizes video and image data from the world’s top professional players to analyze and compare their serves. The system visually examines the results, highlighting similarities and differences between players^17–19^. Figure 3 displays the real-time monitoring of the service training process assisted by AI. Students can observe the technical maneuvers of elite players on the screen, while experienced players can oversee the landing point of the service. AI teaching assistants resolve these queries through big data analysis, presenting the information to students on a large screen in an accessible manner. Simultaneously, the intricate technical procedures can be accelerated and magnified.

AI teaching assistants excel in refining and computing sports indicators, assessing risks and errors in sports information, and collecting real-time sports data during student training. Specialized training sessions undergo continuous monitoring by AI teaching assistants, which involves scrutinizing and comparing the recorded outcomes of each activity with predetermined indicators. In instances where students’ action data deviates from the anticipated indicators, the AI teaching assistants guide corrections through the feedback system. The AI assistant system accurately computes sports technical indicators and promptly communicates them to students. This function enables students to comprehend the shortcomings in their actions and make timely corrections using the sports technical data acquired through sensors and the visual capture system.

### CS-FCM framework based on compressed sensing

While AI has shown success in addressing general issues, it still grapples with overcoming developmental barriers such as interpretability and generalization, particularly in dealing with complex matters. The current limitations of AI include a lack of comprehensive understanding of complexity, and its mathematical foundation faces challenges related to nonlinear issues, resulting in unforeseen outcomes. This uncertainty manifests in the non-uniqueness and instability of complex system learning outcomes and the non-repeatability and unpredictability of system dynamic behavior discovery^20,21^. The primary challenges arising from nonlinear issues in complexity include the scientific description complexity of big data, structural complexity induced by the nonlinear correlation of primary components in complex systems, and evolutionary complexity stemming from the unpredictable regulation of these systems’ behaviors^22–24^. Multi-collinearity is observed in a set of independent variables when, although these variables are not precisely equal to zero when linearly combined with other independent variables, there exists a significant degree of inter-correlation among them. The regression coefficient’s variation is relatively high due to numerous collinearities. Even though the coefficient remains an unbiased estimate, a result with considerable volatility often deviates from reality.

As a tool for knowledge representation and reasoning, FCM depicts a system in a manner akin to human thought processes. Expert knowledge and practical information distilled from data are amalgamated through rule-based structures, emphasizing causal links and graph structures to express knowledge. The predominant focus of FCM researchers lies in implementing and learning techniques, structure expansion methods, and analysis techniques to facilitate decision support across various domains. The framework introduced in this study, CS-FCM, is founded on compressed sensing principles and is applied to the learning of large-scale FCM from time series data.

Compressed sensing, a signal processing technique, adeptly acquires and reconstructs signals by resolving linear systems with limited information. Specifically, compressed sensing aims to reconstruct the vector $X \in R^{N}$ from the linear measurement $Y$ about $X$:

1 $$Y = \phi \times X$$

The more general form can be expressed as Eq. (2).

2 $$Y = \phi \times X + \varepsilon$$

In Eq. (2), $\phi$ indicates the $M \times N$ matrix, and $\varepsilon$ denotes the noise. This problem can be formulated as an $L_{0}$-regularization problem described as Eq. (3).

3 $$\mathop {\min }\limits_{X} \left\| X \right\|_{0} \, s.t. \, Y = \phi X$$

Here, $\left\| X \right\|_{0}$ signifies the $L_{0}$ norm, and $X$ means the number of non-zero elements in v.

$L_{1}$ regularization poses a convex optimization problem, and precise signal reconstruction can be attained by solving the following convex optimization problem:

4 $$\mathop {\min }\limits_{X} \left\| X \right\|_{1} \, s.t. \, Y = \phi X$$

To apply compressed sensing to FCM learning tasks, the problem is initially decomposed into learning local connections for each node. Node *i* can be obtained by:

5 $$\psi^{ - 1} \left( {C_{i} \left( {t + 1} \right)} \right) = \sum\limits_{j = 1}^{N} {w_{ji} C_{j} \left( t \right)}$$

6 $$Y_{i} = \phi \times W_{i}$$

Here, $\psi^{ - 1}$ refers to the inverse function of $\psi$. $\phi$ is determined by the state $C_{j} \left( t \right)$ of the node; $W_{i}$ signifies the weight vector between the *i*th node and all nodes.

To learn the sparse structure from all nodes to node *i*, the following $L_{1}$ regularization problem can be established according to Eq. (7).

7 $$\mathop {\min }\limits_{{W_{i} }} \left\| {W_{i} } \right\|_{1} \, s.t. \, Y = \phi W_{i}$$

To solve Eq. (7), a compressed sensing algorithm based on $L_{1}$ regularization optimization is adopted, and the problem can be defined as Eq. (8).

8 $$\mathop {\min }\limits_{{u,W_{i} }} \sum\limits_{j} {u_{j} } \, s.t. \, Y = \phi W$$

The solution to Eq. (8) involves the use of the primal–dual path tracing interior point method. Initially, it is essential to set dual variables corresponding to $\lambda_{{u_{1} ;j}}$ and $\lambda_{{u_{2} ;j}}$ in Eqs. (9) and (10):

9 $$f_{{u_{1} ;j}} = w_{ij} - u_{j} \le 0$$

10 $$f_{{u_{2} ;j}} = - w_{ij} - u_{j} \le 0$$

The Newton iteration is iterated through the primal–dual algorithm until the proxy dual gap, denoted as $\eta = - f^{T} \lambda$ is reduced below the given tolerance. The maximum iteration is set to 10,000, and the implementation process of CS-FCM unfolds as follows: (1) Initialize $i \leftarrow 1$, and obtain $\phi$ and $Y$. (2) Formulate an objective function for node *i*. (3) Resolve Eq. (1) using the standard interior point method. (4) Update $i \leftarrow i + 1$ n. (5) If ($i > N$), the algorithm halts; otherwise, proceed to step 2.

The least square method and *L*_1_ regulation are incorporated into the FCM weight learning problem. The weight matrix of the FCM is derived by minimizing the error through the least square method, effectively minimizing noise interference. Simultaneously, the minimization of the L_1_ regulation of the weight addresses the challenge of ensuring sparsity in the weight matrix, a limitation in the current learning methods. The nonlinear Hebbian algorithm, a widely employed unsupervised learning method in system weight learning, is utilized. To enhance learning efficiency and model accuracy, the connection weight is adjusted by taking the product of the state values of the causal node and the outcome node linked with the weight without employing additional constraints. As the number of layers updated by Hebb increases, the classification accuracy of the network gradually diminishes, although the accuracy remains superior to the untrained random initialization state. Anti-Hebb plasticity learning diminishes the weight of asynchronously activated neuron connections, with softmax assigning values based on the asynchronous activation value. Consequently, the neuron connections exhibiting poorer synchronization experience higher updating weights.

### Time series prediction framework based on high-order FCM

Neural networks, graph theory, and other topics related to FCM have been extensively applied across various industries, evolving into a prominent research area in the field of AI due to their robust intuitive expression and reasoning capabilities^25–27^. The Fuzzy C-means clustering, also integral to the process of information granulation, typically facilitates the acquisition of core representations by concept nodes. Subsequently, the FCM’s training transforms into an optimization problem for the weight matrix^28^.

In the self-encoder’s output, the middle hidden layer is extracted from feature *X*, and this extracted feature can effectively reconstruct the original feature. In essence, the middle layer accomplishes the abstraction of the original data, serving as an alternative representation. Two structural aspects define the hidden layer in the middle: (1) Dimension reduction, characterized by fewer hidden layer nodes than input layer nodes. (2) Sparsity, involving the addition of sparsity constraints to neurons within the hidden layer.

**Figure 4:**
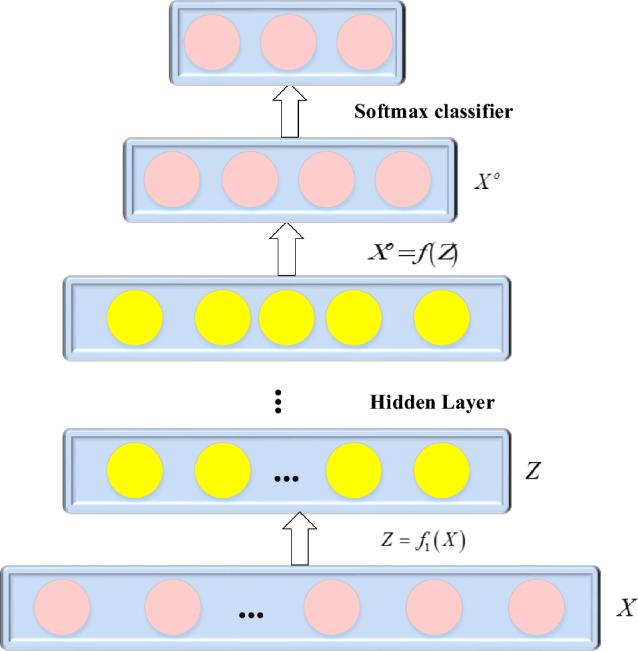
Structure of SAE.

This study uses a Sparse Autoencoder (SAE) to reconstruct the input from its coding representation. The unsupervised learning algorithm is applied to train SAE, addressing the limitation of the prediction model framework based on FCM falling into local optimization. The structure of SAE is depicted in Fig. 4, with each map containing *k* feature nodes. The SAE output is denoted by $X^{o}$, while $W_{S} = \left\{ {W_{1} ,W_{1}^{o} } \right\}$ and $b_{S} = \left\{ {b_{1} ,b_{1}^{o} } \right\}$ respectively represent the weight and bias in SAE^29–31^. Initially, the input data undergoes precise mapping by SAE to obtain a concealed representation. The predicted value of the original time series is then calculated by combining the learned features with the output of the High-order Fuzzy Cognitive Map (HFCM). Granular time series are incorporated to construct the HFCM model, capturing the fundamental properties of time series and ensuring the model’s interpretability.

Each SAE characteristic node is considered a node in the HFCM, ensuring an equal count of characteristic nodes and nodes in the HFCM. The calculation of the node state value in HFCM is elucidated in Eq. (11):

11 $$H\left( t \right) = \varphi \left( {Z\left( t \right)W_{2} + b_{2} + \left[ {Z\left( {t - 1} \right)\left| {...} \right|Z\left( {t - L + 1} \right)W_{x} } \right]} \right)$$

Here, $\left[ {.\left| . \right.} \right]$ stands for vertical vector connection. $Z$ and $H$ are used to calculate the output of SAE-FCM using Eq. (12):

12 $$Y\left( t \right) = \left[ {Z\left( t \right)\left| {H\left( t \right)} \right.} \right]W_{3} = BW_{3}$$

In Eq. (12), $W_{3} \in R^{2k \times 1}$, and the training of $W_{3}$ is a linear task, and its calculation can be completed by ridge regression:

13 $$W_{3} = \left[ {B^{T} B + \lambda I} \right]^{ - 1} B^{T} Y$$

In Eq. (13), $\lambda$ and $I$ represent a constant and an identity matrix, respectively.

For enhanced prediction accuracy of the SAE-HFCM model, fine-tuning is employed to adjust the model parameters. Should the targeted training accuracy remain elusive post fine-tuning, the process is reiterated. The training algorithm flow of the SAE-HFCM model is delineated in Table 2.

**Table 2:** The training algorithm flow of the SAE-HFCM model.

Algorithm: SAE-HFCM model training algorithm
Input: $\varepsilon$: training accuracy of SAE-FCM; $\left\{ {X,Y} \right\}$: training data; Output: $W_{1}$, $W_{2}$, $W_{3}$, $W_{x}$, $b_{1}$ and $b_{2}$
1: Get $Z$ from input data $X$ with SAE, and record $W_{1}$ and $b_{1}$
2: Randomly initialize $W_{2}$, $W_{x}$ and $b_{2}$, and get $Z$ from $H$
3: Calculate $W_{3}$ by ridge regression
4: Fix $W_{1}$, $b_{1}$ and $W_{3}$, and update $W_{2}$, $W_{x}$ and $b_{2}$
5: **while** Training accuracy < $$\varepsilon$$$$\square$$ **do < **
6: Update $$W_{1}$$ and $$b_{1}$$
7: Update $$W_{2}$$, $$W_{x}$$ and $$b_{2}$$
8: Update $$W_{3}$$
9: **end while**
10: **return** $$W_{1}$$,$$W_{2}$$, $$W_{3}$$, $$W_{x}$$, $$b_{1}$$ and $$b_{2}$$

This study assesses the effectiveness of the SAE-HFCM model using four datasets—Sunspot, S&P500, Radio, and Lake—each exhibiting diverse features. The Sunspot dataset, tracking sunspot counts from 1700 to 1987, demonstrates strong temporal correlation and periodicity, posing a challenge for time series predictions. The S&P500 dataset, encompassing the daily starting prices of the S&P 500 index from June 1, 2016, to June 1, 2017, is marked by complexity and nonlinearity due to various influencing factors in the stock market. The Radio dataset, spanning radio broadcasts in Washington, DC, from May 1934 to April 1954, is characterized by significant nonlinearity and complexity, making traditional linear models less effective. The Lake dataset covers monthly water level measurements of Lake Erie from 1921 to 1970, containing multivariate information and spatial correlation, necessitating using geographic information system (GIS) models for predictions. For statistical significance testing of datasets, the Lake dataset is exemplified. It includes geographical location, depth, water temperature, and weather information. Four models are established to explore the impact of these factors on lake water quality: a full variable model and three univariate models (each containing only one independent variable). Initially, the statistical significance of the full variable model is tested using the F-test and *P* value. Results indicate that all independent variables in the full variable model significantly influence lake water quality (*P* values are all less than 0.05). Subsequently, the coefficient significance test is conducted, affirming the reliability of all coefficient estimates (*p *values of the *T* test are less than 0.05). Following this, three univariate models are tested for statistical significance through univariate regression analysis and *P* value assessment, revealing that independent variables in all univariate models significantly impact lake water quality (*P* values are less than 0.05).

The model selection process employs the grid search approach, segmenting the standard time series data into three subsets: the training set (70% of the total data), validation set (10%), and test set (20%). During training, the model learns historical data patterns from the time series to inform future predictions. The validation set is utilized to fine-tune model parameters and select the optimal model. Finally, the test set evaluates the model’s overall performance. The optimal prediction model is chosen, and prediction accuracy is assessed using both the validation set and the test set. SAE processes the training data to derive time series characteristics, with the number of nodes in HFCM aligning with the hidden units in SAE. To gauge performance, a comparative analysis is conducted between SAE-HFCM and the Autoregressive Model Per Scale (AR)^32^ and ANN^33^. The difference in performance on the Root Mean Square Error (RMSE) index is specifically examined.

## Results and discussion

### Convergence and stability analysis of CS-FCM

**Figure 5:**
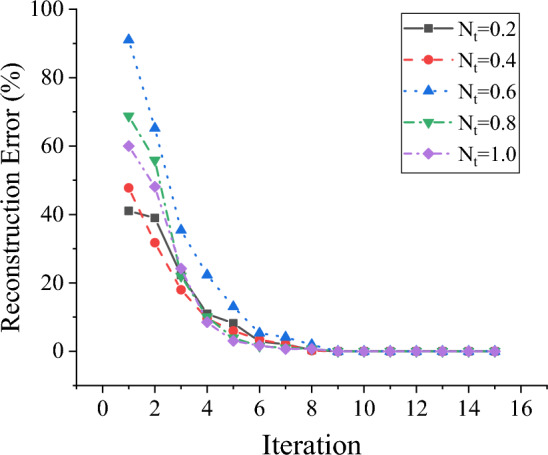
Convergence and stability of CS-FCM with data density of 20%.

**Figure 6:**
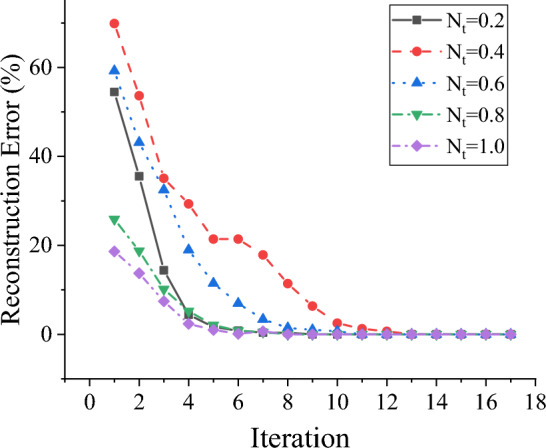
Convergence and stability of CS-FCM with data density of 40%.

In this study, the convergence and stability of CS-FCM are examined, and the outcomes of experiments conducted on FCM with densities of 20% and 40% are depicted in Figs. 5 and 6. The experiments involve 100 nodes, with varying *N*_1_ values set at 0.2, 0.4, 0.6, 0.8, and 1.0, where *N*_1_ represents the total data length divided by the number of nodes. As illustrated in Fig. 5, for FCM with a 20% density and different data lengths, CS-FCM demonstrates remarkable efficiency by converging to a stable point with a reconstruction error below 10^–3^ in just 15 iterations. Figure 6 further reveals that, for FCM with a 40% density, CS-FCM achieves stability after 17 iterations. Hence, CS-FCM exhibits robust convergence and stability across varying densities and data lengths. Furthermore, the study explores the scalability of CS-FCM by testing it on an FCM with 1000 nodes. Despite a decrease in accuracy with an increased number of nodes, CS-FCM demonstrates its capability to learn large-scale FCM structures.

As the number of nodes within the FCM increases while maintaining the same density, the algorithm is required to establish more connections between them. Despite a decrease in accuracy with an expanding number of nodes, CS-FCM can effectively learn large-scale FCM structures. This underscores the model’s proficiency in connection learning, enabling it to navigate complex network structures. It is noteworthy that these findings, although derived from a specific dataset, highlight the model’s adaptive learning strategy, allowing the model to autonomously adjust to diverse problem scenarios. Moreover, leveraging compressed sensing technology empowers the model to handle large-scale data efficiently without additional data annotation. This emphasizes the model’s robust generalization capability, making it applicable across multiple problem scenarios. Comparative analysis of FCM performance under varied density conditions enhances comprehension of the algorithm’s limitations and potential and opens avenues for refining and optimizing the FCM algorithm to better suit diverse and intricate data network environments.

### Performance evaluation of the SAE-HFCM model in a complex system

**Figure 7:**
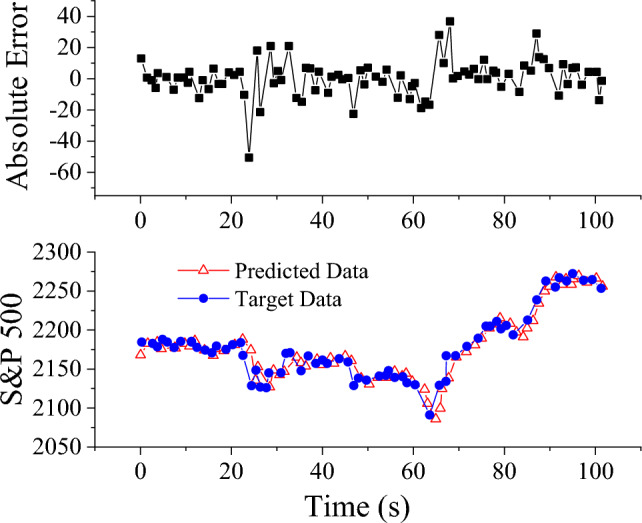
Prediction curve and error curve based on SAE-HFCM model on S&P500 data set.

The hyperparameters of the comparative models are determined through rigorous experimentation. The RMSE results for the SAE-HFCM model and other methodologies proposed here are ultimately obtained through extensive trials and summarized in Table 3 across four datasets. Notably, the results indicate that, across all datasets, the SAE-HFCM model introduced in this study surpasses both AR and ANN. The prediction curve and error curve of the SAE-HFCM model are illustrated on the test set to provide a visual representation. Figure 7, utilizing the time series of the S&P500 dataset as an example, showcases that the SAE-HFCM model has achieved remarkable accuracy. It is noteworthy that the primary source of current errors is the sudden changes in the time series. This observation aligns with the conclusions of Stefenon et al. (2021)^34^, corroborating the validity and effectiveness of this study.

**Table 3:** RMSE comparison results of different models on four data sets.

Data set	SAE-HFCM	AR	ANN
Sunspot	17.392	35.262	19.900
0S&P500	11.891	17.896	17.698
Radio	0.488	0.904	0.653
Lake	0.358	0.392	0.402

## Conclusion

This study endeavors to investigate a sports education teaching model guided by high-order complex networks, wherein an AI assistant delivers intelligent assistance in tennis instruction through CS-FCM. CS-FCM, a pivotal component of this system, is integrated into the AI assistant, employing compressed sensing principles. This innovative approach encompasses a large-scale FCM learning framework, specifically named CS-FCM. The framework is scrutinized using experimental data with FCM densities of 20% and 40%, providing valuable insights into the efficacy of the system. The SAE is trained utilizing unsupervised learning techniques to address the deficiency in the architecture of the FCM-based prediction model, which is susceptible to local optimization. Utilizing the acquired features in conjunction with the output from HFCM, the predicted values of the original time series are determined. The research findings reveal that, for an FCM density of 20%, CS-FCM attains a stable point within 15 iterations, with a reconstruction error below 10^–3^. In the case of an FCM density of 40%, stability is achieved after 17 iterations. These results unequivocally demonstrate the superior performance of CS-FCM in large-scale FCM learning. Despite a decline in accuracy with an increase in node quantity, CS-FCM remains effective in learning large-scale FCM, showcasing the model’s robust capacity in connection learning. Particularly noteworthy is its excellent performance in handling complex network structure data. Moreover, the RMSE results of the SAE-HFCM model on four datasets significantly surpass those of traditional AR and ANN models. The experiments, illustrated by the time series of the S&P500 dataset, highlight the exceptional predictive accuracy of the SAE-HFCM model on the test set. It demonstrates notable adaptability to abrupt changes in time series. In conclusion, this study validates the feasibility and effectiveness of CS-FCM in an intelligent sports education system. Furthermore, with the introduction of the SAE-HFCM model, the performance of the FCM algorithm in time series prediction is enhanced. The research results provide robust support for intelligent teaching systems and offer valuable insights for intelligent-assisted teaching in various domains. The approach delineated in this study exhibits certain limitations, primarily rooted in its dependence on automatic feature extraction and representation learning. This reliance may potentially constrain its performance under specific circumstances. Subsequent research endeavors could delve into the exploration of more sophisticated techniques in data processing and feature engineering. This exploration aims to effectively capture the intricate and nuanced properties inherent in physical training data, thereby advancing the robustness and versatility of the proposed methodology.

### Supplementary Information

Supplementary Information 1.

Supplementary Information 2.

Supplementary Information 3.

Supplementary Information 4.

Supplementary Information 5.

Supplementary Information 6.

Supplementary Information 7.

Supplementary Information 8.

## Data Availability

All data generated or analyzed during this study are included in this published article (and its Supplementary Information files).
