# Supplementary figures and images for: Physical education teaching mode assisted by artificial intelligence assistant under the guidance of high-order complex network
Source: Sci Rep. 2024 Feb 19;14:4104. doi: 10.1038/s41598-024-53964-7 (PMC10876635; doi:10.1038/s41598-024-53964-7)

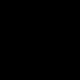

Supplement: Supplementary file 2 — Supplementary Information 2. [file 41598_2024_53964_MOESM2_ESM.vsdx › visio/media/image7.bmp]

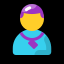

Supplement: Supplementary file 2 — Supplementary Information 2. [file 41598_2024_53964_MOESM2_ESM.vsdx › visio/media/image8.bmp]

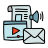

Supplement: Supplementary file 2 — Supplementary Information 2. [file 41598_2024_53964_MOESM2_ESM.vsdx › visio/media/image5.png]

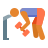

Supplement: Supplementary file 2 — Supplementary Information 2. [file 41598_2024_53964_MOESM2_ESM.vsdx › visio/media/image1.png]

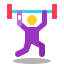

Supplement: Supplementary file 2 — Supplementary Information 2. [file 41598_2024_53964_MOESM2_ESM.vsdx › visio/media/image2.png]

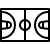

Supplement: Supplementary file 2 — Supplementary Information 2. [file 41598_2024_53964_MOESM2_ESM.vsdx › visio/media/image6.png]

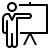

Supplement: Supplementary file 2 — Supplementary Information 2. [file 41598_2024_53964_MOESM2_ESM.vsdx › visio/media/image4.png]

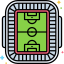

Supplement: Supplementary file 2 — Supplementary Information 2. [file 41598_2024_53964_MOESM2_ESM.vsdx › visio/media/image3.png]

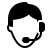

Supplement: Supplementary file 3 — Supplementary Information 3. [file 41598_2024_53964_MOESM3_ESM.vsdx › visio/media/image4.png]

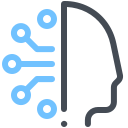

Supplement: Supplementary file 3 — Supplementary Information 3. [file 41598_2024_53964_MOESM3_ESM.vsdx › visio/media/image1.png]

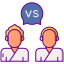

Supplement: Supplementary file 3 — Supplementary Information 3. [file 41598_2024_53964_MOESM3_ESM.vsdx › visio/media/image5.png]

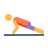

Supplement: Supplementary file 3 — Supplementary Information 3. [file 41598_2024_53964_MOESM3_ESM.vsdx › visio/media/image3.png]

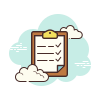

Supplement: Supplementary file 3 — Supplementary Information 3. [file 41598_2024_53964_MOESM3_ESM.vsdx › visio/media/image2.png]

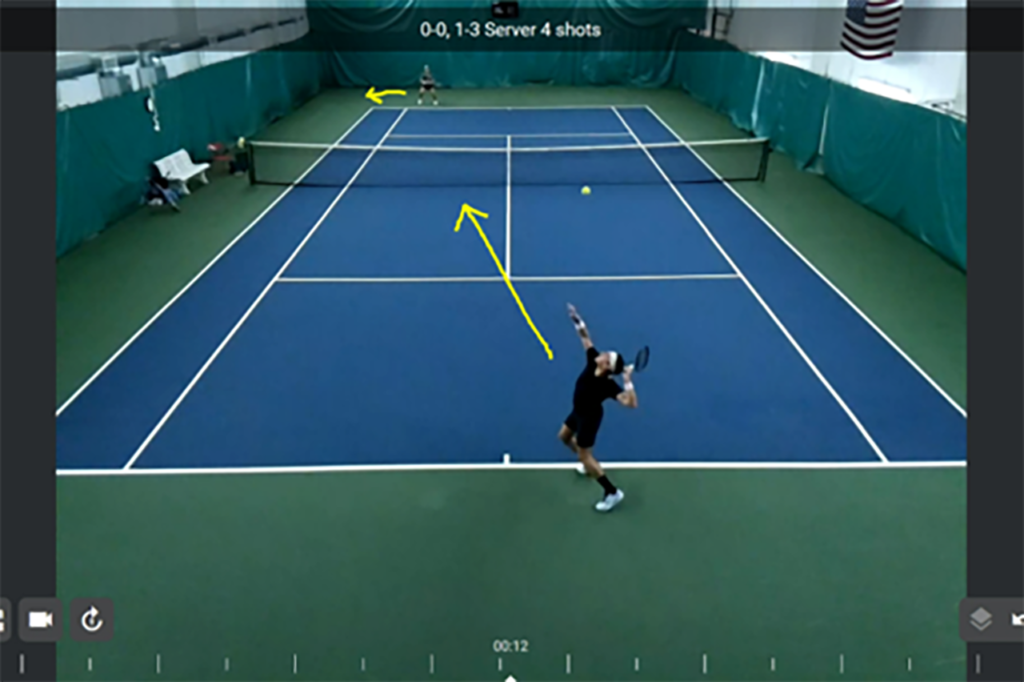

Supplement: Supplementary file 4 — Supplementary Information 4. [file 41598_2024_53964_MOESM4_ESM.vsdx › visio/media/image1.png]
